# Correction: An efficient one pot *ipso*-nitration: structural transformation of a dipeptide by N-terminus modification

**DOI:** 10.1039/c9ra90053e

**Published:** 2019-07-10

**Authors:** Rajib Sarkar, Krishnendu Maji, Debasish Haldar

**Affiliations:** Department of Chemical Sciences, Indian Institute of Science Education and Research Kolkata Mohanpur India deba_h76@yahoo.com deba_h76@iiserkol.ac.in +913325873020 +913325873119

## Abstract

Correction for ‘An efficient one pot *ipso*-nitration: structural transformation of a dipeptide by N-terminus modification’ by Rajib Sarkar *et al.*, *RSC Adv.*, 2015, **5**, 59570–59575.

The authors wish to clarify a few sentences by stating that the major product in the *ipso*-nitration reaction is probably the phenol 3 (Scheme 1). The *ipso*-nitration reaction happened in TFA, NaNO_2_ and H_2_SO_4_, which are comparatively drastic conditions for a peptide. Initially the yield of target compound 2 was 40%. As we had a single crystal structure matching the structure of compound 2, and the structure exactly matched with the mass spectroscopy data, we did not analyze the remaining 60% of the reaction product, which was likely phenol 3. Moreover, phenol 3 has the same formula and mass as our target compound 2. We have been misled by this fact and did not analyze the reaction mixture further. The moderate yields may indeed indicate that the other product (phenol 3) could have been present which we have missed.

**Scheme 1:**
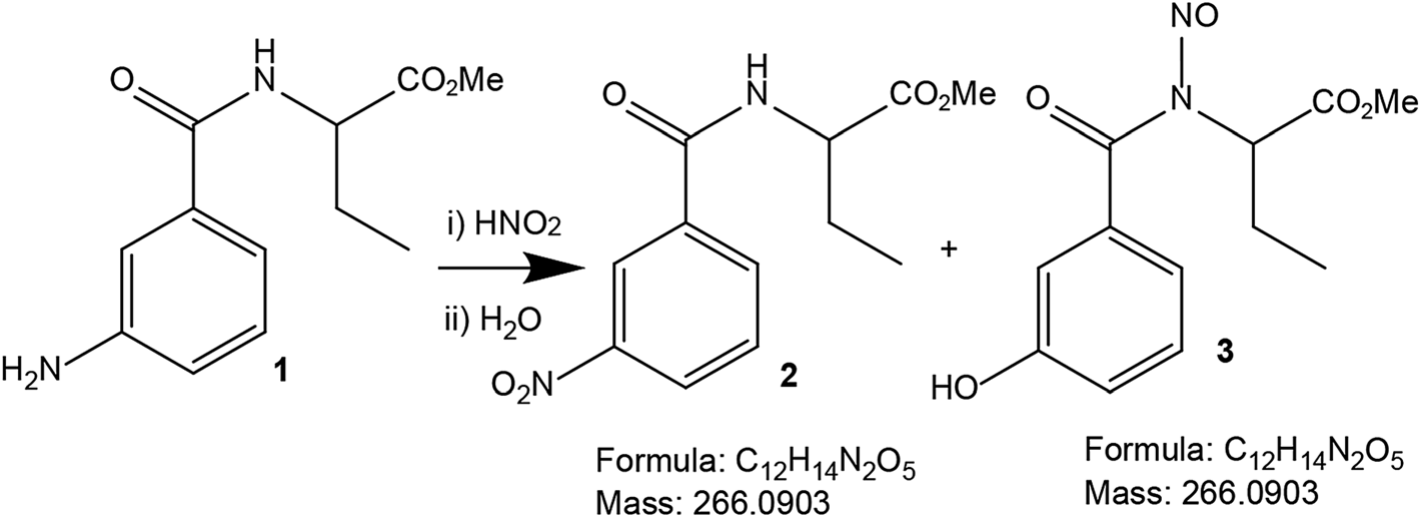
The schematic presentation of possible products from the *ipso*-nitration of peptide 1. Both products 2 and 3 have the same chemical formula and exact mass.

Ref. 14 should also be updated to include more relevant literature reports of *ipso*-nitration (ref. 14d-h in the article, presented here as ref. 1*a*–*e*). In 1939, E. B. Starkey reported the replacement of an amine group by a nitro group through a diazotization reaction.^1*a*^ In 1947, Hodgson and co-workers reported the replacement of a diaxonium by a nitro group.^1*b*^

Singh and co-workers proposed a radical mechanism (Scheme 2) for the *ipso*-nitration reaction.^1*e*^ Therefore, the mechanism we originally proposed in the article may not be correct, as we are unable to rule out the possibility that the reaction occurs *via* a radical mechanism.

**Scheme 2:**
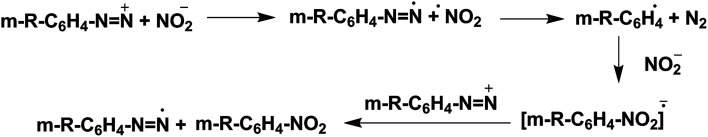
The schematic of a radical mechanism adopted from *Tetrahedron Lett.*, 1982, **23**, 5191.

The authors apologise for these errors and are indebted to an *RSC Advances* Board member and the editorial team for the exchange of information and the discussion regarding this corrigendum.

The Royal Society of Chemistry apologises for these errors and any consequent inconvenience to authors and readers.

## Supplementary Material
